# MEMS strapdown inertial attitude measurement system using rotational modulation technology

**DOI:** 10.1371/journal.pone.0298168

**Published:** 2024-02-13

**Authors:** Wei Sun, Heng Huang, Peilun Sun, Wei Ding

**Affiliations:** School of Geomatics, Liaoning Technical University, Fuxin, China; Guizhou University of Finance and Economics, CHINA

## Abstract

Attitude determination involves the integration of methodologies and systems for estimating the time varying attitude of moving objects. Strapdown Inertial Attitude Measurement System (SIAMS) is among the most widely used navigation systems. The development of cost effective Micro Electro Mechanic System (MEMS) based inertial sensors has made attitude measurement system more affordable. However, MEMS sensors suffer from various errors that have to be calibrated and compensated to get acceptable attitude results. Given the auto-compensation of inertial sensor bias in rotation error modulation, the objective of this paper is to develop a MEMS-based rotary SIAMS, in which the significant sensor bias is automatically compensated by rotating the IMU, to offer comparable performance with respect to a tactical-grade Inertial Measurement Unit (IMU). With the analysis of the relationship between the MEMS error and misalignment, a MEMS calibration model is derived, and a combined calibration method of multi position rotation is applied to estimate the deterministic sensor errors such as bias, scale factor, and misalignment. Simulation and experiment results indicate that the proposed method can further modulate and compensate the MEMS errors, thereby improving the MEMS attitude accuracy.

## 1. Introduction

Since the development of navigation and positioning technology, the best estimate of the moving vehicle’s attitude can be obtained using an attitude measurement system, which includes the Global Navigation Satellite System (GNSS) and Inertial Navigation Systems (INS) [1, 2]. Since GNSS cannot be applied to all environments, the need to find an alternative attitude measurement system is stronger. The emergence of MEMS technology is a promising development. The INS based on MEMS can provide short-term high-precision attitude measurement, but the accuracy cannot be guaranteed in long-term attitude measurement tasks. This is because the MEMS inertial navigation system only uses the accelerometer and gyroscope data provided by IMU for attitude calculation. Long time observation will lead to error accumulation, which will eventually affect the results and generate divergent inertial solutions [3–5]. Therefore, the inertial sensor error must be compensated to improve the performance of MEMS inertial navigation.

Although the commonly used multi-system integrated attitude estimation method based on filtering can provide good attitude measurement results, the cost is higher than that of a single navigation system, and the introduction of more state variables makes the model structure more complex. Therefore, this paper studies the rotation error modulation technology which only considers the inertial reference information. It only needs to move the IMU on the rotating platform according to the predetermined rotation scheme, and improve the attitude accuracy by compensating the navigation error caused by the inertial sensor bias in the entire rotation cycle [6–8]. This technology is applied to a MEMS-based rotating Strapdown Inertial Attitude Measurement System (SIAMS).

Reference [9] compares and analyzes three types of rotation schemes, and finds that the single-axis reciprocating rotation scheme has the best effect. However, the error characteristics of different rotation schemes are not analyzed in depth. Reference [10] analyzes the error characteristics of different rotation schemes and compensates the error of single-axis rotation scheme, but does not consider the requirements of actual use environment. Reference [11] designs a new rotation scheme suitable for high dynamic environment, but the equipment cost is high and the universality is low.

**Fig 1:**
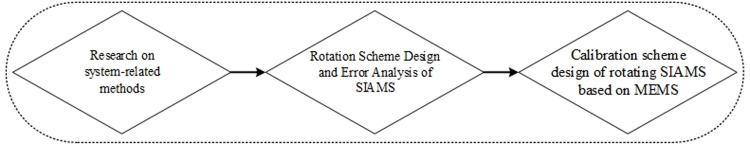
Schematic diagram of design scheme.

Therefore, this paper discusses the single-axis reciprocating rotary modulation technology, analyzes the error characteristics of this scheme, and proposes a combined calibration method of multi position rotation without introducing external information to provide the same attitude measurement accuracy as a tactical-grade IMU. The flow chart of this study is shown in the Fig 1 and the contributions of this article are:

- A MEMS-based rotation SIAMS method is proposed in this paper, which uses a single axis reciprocating rotation scheme to rotate IMU. Due to the periodic rotation of the system, the divergent inertial bias is converted into a periodic signal, which is eliminated during the rotation period. Therefore, the attitude error caused by constant bias of inertial elements is compensated automatically.
- Since the rotation modulation technology cannot restrain the attitude error caused by the scale factor and axis misalignment of the gyro, a combined calibration method of multi position rotation is proposed to solve this problem. The attitude estimation accuracy of MEMS can be further improved by this method.

## 2. Backdrop and devices

### 2.1. Rotary strapdown inertial attitude measurement system

Inertial attitude measurement system is a device to decide the attitude of an object by using the observation value supplied by the IMU attached to the object to be decided. The angular motion of the object to be decided in the inertial coordinate system can be described by the blended observation value of the accelerometer and gyroscope carried by the IMU, and its attitude can be calculated according to the preliminary state value [12–14]. The traditional inertial attitude measurement technology is a technology that installs inertial sensors on a stable platform and mechanically isolates the object to be decided from the rotating motion unit, which is called platform inertial attitude measurement system. In recent years, SIAMS has eliminated the mechanical complexity of the platform system by rigidly connecting the sensor to the object to be decided. On the other hand, SIAMS is smaller in size, lighter in weight, and more reliable in measurement values, so it gradually replaces the application of the platform inertial measurement system in most scenarios [6].

**Fig 2:**
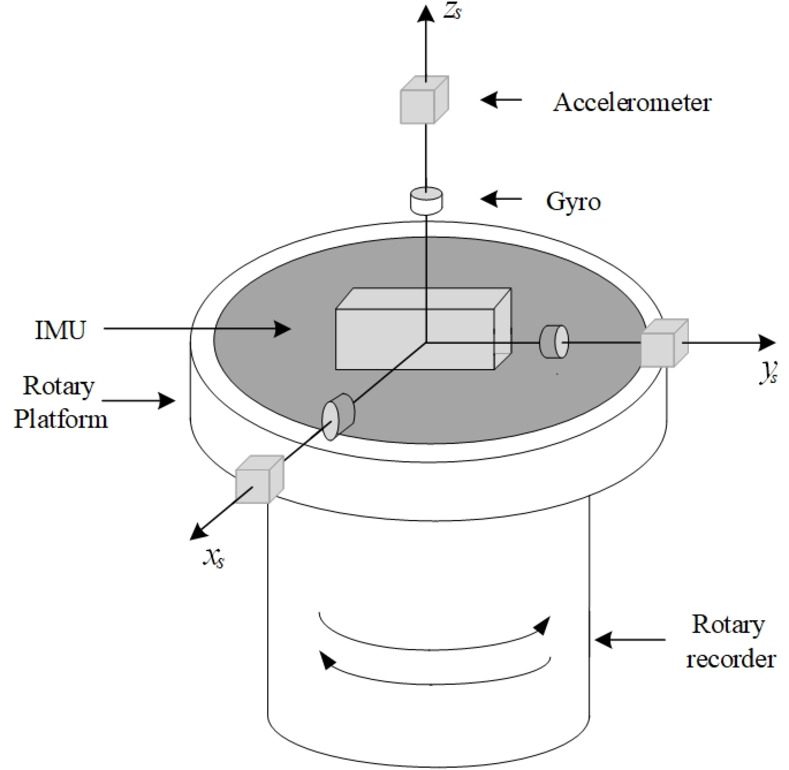
Structure schematic of a typical rotary SIAMS.

Rotational strapdown inertial attitude measurement technology has robust autonomy. It can weaken or compensate the influence of inertial sensor error without the usage of external information [15, 16]. As shown in Fig 2, The encoder installed on the rotating platform is embedded with the rotation configuration of SIAMS, which can measure the angle of IMU and the frame of the object to be measured. Due to the periodic rotation of IMU, the divergent inertial bias is converted into a periodic signal, which is eliminated during the rotation period. This is the core idea of rotary modulation technology.

**Fig 3:**
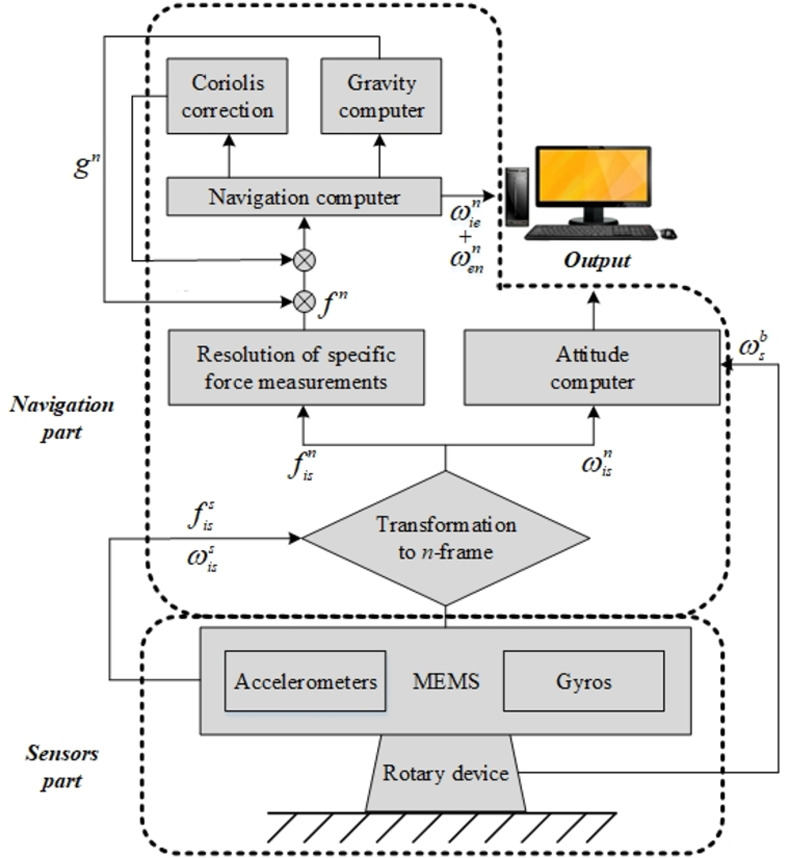
Mechanization of the rotary SIAMS.

The SIAMS fit into the rotary modulation technology uses the mechanization algorithm based on the classic strapdown inertial navigation system [17], and makes some changes to the characteristics of MEMS sensors, as shown in Fig 3. First, a new inertial navigation frame is defined, i.e., the sensor frame (*s*-frame) in the figure. The *s-*frame takes the origin of the navigation frame (*n*-frame) as the origin, consists of three orthogonal sensitive axes, and changes according to the real-time attitude provided by IMU. Unlike the traditional strapdown system, SIAMS uses the *s*-frame to obtain IMU data. Because the body frame and IMU are not aligned due to rotation, a direction cosine matrix is used to convert *s*-frame data to *n*-frame data. At the same time, the relationship between *s*-frame and the frame of the object itself (*b*-frame) should also be considered when calculating the attitude of the object to be determined [18].

### 2.2. MEMS rotary SIAMS error modulation

**Fig 4:**
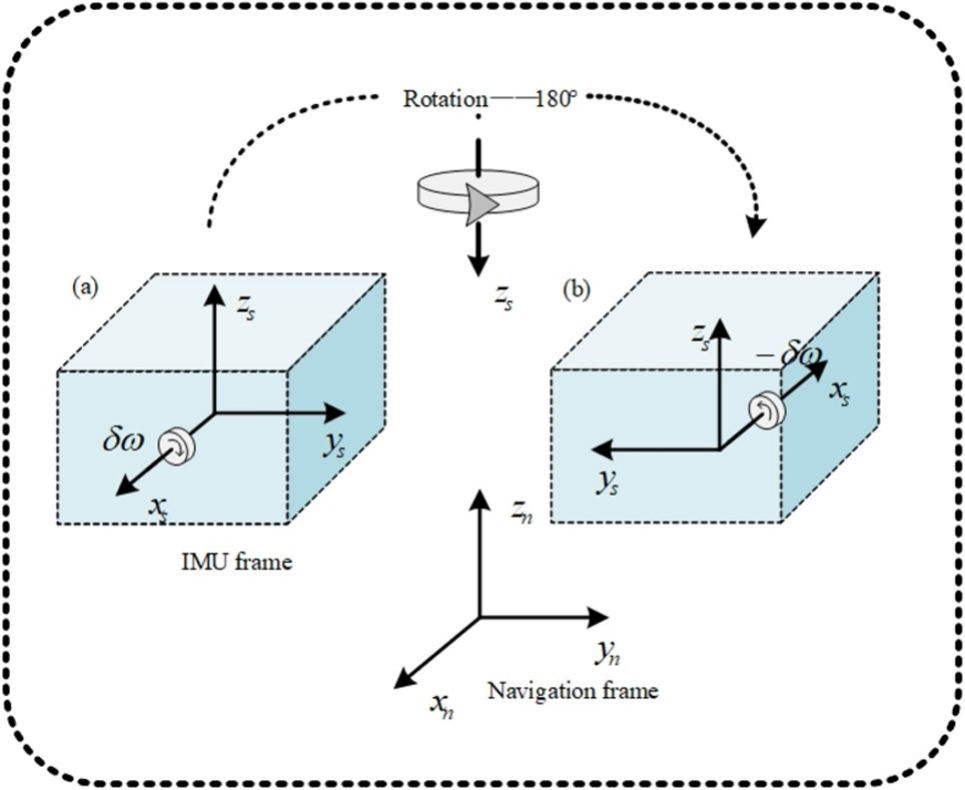
IMU rotational motion.

SIAMS calculates the current position information through the three-dimensional distance provided by the previous position. Therefore, the attitude solution error of the object to be determined will accumulate with the increase of time. This is because the increase of time will cause the drift offset error of the accelerometer and gyroscope to increase. To solve this problem, the rotating SIAMS automatically compensates the error by rotating the IMU periodically to convert the sensor bias into a periodic signal. In the following, we will introduce the rotary modulation technology in detail. Fig 4 shows the principle of rotary modulation to weaken or eliminate errors. The IMU rotates around the *b*-frame coordinate axis according to the set track. As shown in Fig 4(A), error *δω* exists in gyroscope output information contained in *x*_*s*_ axis of IMU coordinate system. On this premise, as shown in Fig 4(B), The phase under the navigation frame reverses through rotation, so the IMU rotates 180 degrees around the *z*_*s*_ axis in a horizontal attitude, and its error changes -*δω* is eliminated. It should be noted that there is a constraint condition for the error weakening effect caused by IMU rotation, i.e., the object to be determined must meet the requirement that the IMU always keep level with its own attitude. In this state, the drift bias error caused by the accelerometer and gyroscope placed on the non-rotating axis of the *b*-frame is alleviated through the rotating motion, and finally the attitude accuracy of the object to be determined is improved.

This paper will research and develop a low-cost single axis rotation SIAMS based on MEMS with high reliability and simple structure. Sensonor’s STIM202 MEMS gyroscope, Colibris’s MS8010-D MEMS accelerometer, single-axis turntable and angle encoder are used for the research and development of the methods proposed in the paper. Table 1 shows the error parameters of MEMS sensors. The rotating platform of IMU is a single-axis turntable, and the angle between the frame of the object to be determined and the IMU frame is provided by the angle encoder.

**Table 1:** Error parameters of inertial elements.

Sensor performance	Gyroscope	Accelerometer
Dimensions	3 axes	3 axes
Full Scale	±400 deg/s	±10 m/s^2^
Linearity	0.1% of FS	0.2% of FS
Bias stability	0.5 deg/h	0.0075 m/s^2^
Scale Factor stability	-	300 ppm
Noise	0.05 deg/s/Hz^1/2^	0.002 m/s^2^/Hz^1/2^
Bandwidth	262 Hz	200 Hz

In this research, the rotating error modulation technology is used to reduce the significant sensor error of the inertial measurement unit, and the following work is contributed to achieve the goal of using this technology to improve the attitude accuracy of the MEMS based rotating SIAMS:

- *Rotation system modeling and error analysis*
- *Combine calibration method of multi position for MEMS-based rotary SIAMS*

The performance and manufacturing cost of the system are closely related to the rationality of the construction of the rotation model. This is because the sensor errors are diverse and have cross-correlation property with the navigation system. The improper rotation scheme will not only not weaken the error, but also even magnify the error or add new error items. Based on the results of error analysis, this paper proposes a scheme of single axis rotation (also called indexing rotation).

## 3. Rotation system modeling and error analysis

### 3.1. Reciprocating rotation

**Fig 5:**
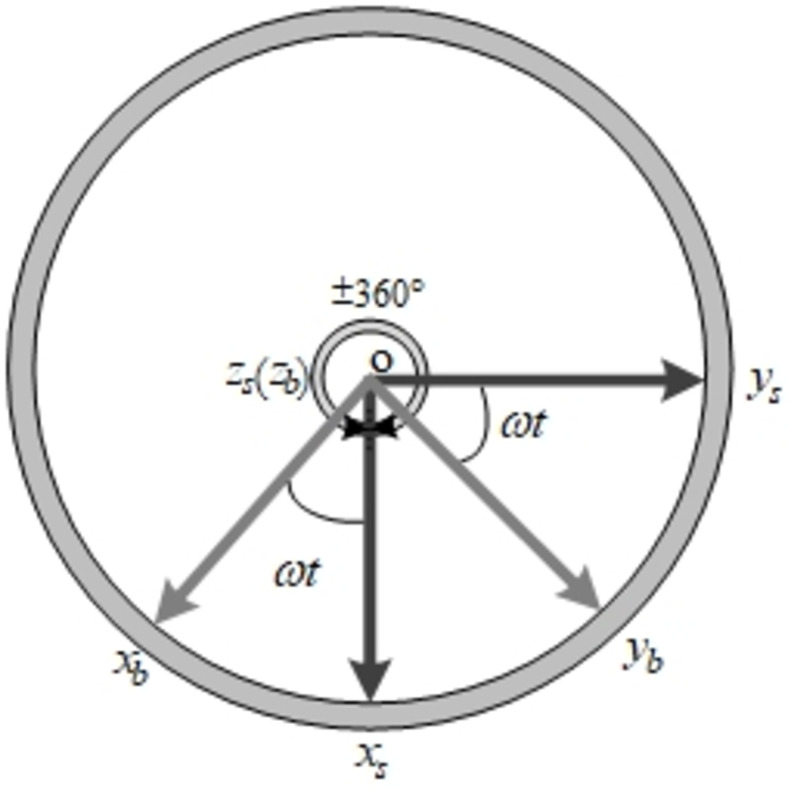
IMU indexing rotation.

The design of reciprocating rotation scheme makes SIAMS get rid of the requirement of slip ring. As shown in Fig 5, the system rotates 360 degrees counterclockwise (positive) around the *z*_*s*_ axis at a constant rotation rate ω, and then rotates 360 degrees clockwise (negative) around the *z*_*s*_ axis, forming a complete reciprocating rotation cycle. At the same time, it is assumed that the body frame and the navigation frame are aligned to simplify the error analysis [19]. At this point, the transformation matrix from the IMU frame to the body frame is:

$$C_{b}^{i}=\left[\begin{array}{ccc} \text{cos}\;\omega t & \text{sin}\;\omega t & 0\\ -\text{sin}\;\omega t & \text{cos}\;\omega t & 0\\ 0 & 0 & 1 \end{array}\right]$$ (1)

It should be noted that, this assumption reduces the complexity of calculation. In practical applications, a set of matrices can be added for the conversion of body frame and navigation frame.

$$C_{n}^{b}=\left[\begin{array}{ccc} \text{cos}\;R\;\text{cos}\;H+\text{sin}\;P\;\text{sin}\;R\;\text{sin}\;H & \text{cos}\;R\;\text{sin}\;H+\text{sin}\;P\;\text{sin}\;R\;\text{cos}\;H & -\text{cos}\;P\;\text{sin}\;R\\ -\text{cos}\;P\;\text{sin}\;H & \text{cos}\;P\;\text{cos}\;H & \text{sin}\;P\\ \text{sin}\;R\;\text{cos}\;H+\text{sin}\;P\;\text{cos}\;R\;\text{sin}\;H & \text{sin}\;P\;\text{cos}\;P\;\text{cos}\;H & \text{cos}\;P\;\text{cos}\;R \end{array}\right]$$ (2)

where $C_{n}^{b}$ denotes the attitude transformation matrix from the navigation frame to the body frame, which is related to the rotation order around the three axes. H, P, and R are the heading, pitch, and roll angles of the carrier.

There are three types of errors to be analyzed, as shown below:

- *Constant Bias*
- *Scale Factor Error*
- *Installation Error (caused by misalignment)*.

#### Constant bias

Eq (3) represents the modulation of constant bias of the navigation frame when the IMU rotates in the forward direction. On the contrary, Eq (4) represents the modulation of constant bias when the IMU rotates in the reverse direction [20].

$$\varepsilon_{+}^{n}=\left[\begin{array}{ccc} \text{cos}\;\omega t & -\text{sin}\;\omega t & 0\\ \text{sin}\;\omega t & \text{cos}\;\omega t & 0\\ 0 & 0 & 1 \end{array}\right]\left[\begin{array}{c} \varepsilon_{x}^{s}\\ \varepsilon_{y}^{s}\\ \varepsilon_{z}^{s} \end{array}\right]=\left[\begin{array}{c} \varepsilon_{x}^{s}\text{cos}\;\omega t-\varepsilon_{y}^{s}\text{sin}\;\omega t\\ \varepsilon_{x}^{s}\text{sin}\;\omega t+\varepsilon_{y}^{s}\text{cos}\;\omega t\\ \varepsilon_{z}^{s} \end{array}\right]$$ (3)

$$\varepsilon_{-}^{n}=\left[\begin{array}{ccc} \text{cos}\;\omega t & \text{sin}\;\omega t & 0\\ -\text{sin}\;\omega t & \text{cos}\;\omega t & 0\\ 0 & 0 & 1 \end{array}\right]\left[\begin{array}{c} \varepsilon_{x}^{s}\\ \varepsilon_{y}^{s}\\ \varepsilon_{z}^{s} \end{array}\right]=\left[\begin{array}{c} \varepsilon_{x}^{s}\text{cos}\;\omega t+\varepsilon_{y}^{s}\text{sin}\;\omega t\\ -\varepsilon_{x}^{s}\text{sin}\;\omega t+\varepsilon_{y}^{s}\text{cos}\;\omega t\\ \varepsilon_{z}^{s} \end{array}\right]$$ (4)

where "+" in subscript represents forward rotation, and "-" in subscript represents reverse rotation, ${\text{[}\varepsilon_{x}^{s}\varepsilon_{y}^{s}\varepsilon_{z}^{s}\text{]}}^{T}$ and ε^*n*^ are respectively representing gyroscopic bias in IMU frame and navigation frame.

Assume that the time of both rotations is *T*. It can be inferred that the complete reciprocating rotation cycle is *T*′ = 2*T*. Eq (5) shows the attitude error caused by gyroscopic constant bias during the complete cycle.


$$\int_{0}^{T^{\prime }}\varepsilon^{n}dt=\int_{0}^{T}\varepsilon_{+}^{n}dt+\int_{T}^{2T}\varepsilon_{-}^{n}dt=\left[\begin{array}{c} 0\\ 0\\ T^{\prime }\nabla_{z}^{s} \end{array}\right]$$ (5)

Reciprocating rotation is similar to one-way rotation. Setting gyroscope bias to periodic signal will not produce attitude error on horizontal plane. However, the gyroscope bias on the rotation axis always exists, so the course (heading) error will accumulate as time increase. Eq (6) analyzes the error caused by accelerometer bias ∇. Based on this, the non-rotating axis bias can be modulated into a periodic signal, and the integral of the entire period is zero. Similar to the gyroscope bias, the accelerometer bias on the rotation axis cannot be eliminated, so it will also lead to the vertical error accumulated over time.


$$\int_{0}^{T^{\prime }}\nabla^{n}dt=\int_{0}^{T}\nabla_{+}^{n}dt+\int_{T}^{2T}\nabla_{-}^{n}dt=\left[\begin{array}{c} 0\\ 0\\ T^{\prime }\nabla_{z}^{s} \end{array}\right]$$ (6)

#### Scale factor error

The scale factor applied to the model has been calibrated, but its characteristics of changing with time and temperature lead to the residual error of the scale factor in the inertial sensor, so the rotary modulation error caused by the residual error cannot be ignored [21]. When the IMU rotates relative to the *z*_*s*_ axis, the rotation data of the earth and IMU are provided by the gyroscope. Eq (7) gives the theoretical output of the gyroscope in the IMU frame relative to the inertial frame, while Eq (8) describes the error caused by the scale factor [22].

$$\omega_{is}^{s}=\left[\begin{array}{c} \omega_{isx}^{s}\\ \omega_{isy}^{s}\\ \omega_{isz}^{s} \end{array}\right]=\left[\begin{array}{c} \omega_{ie}\text{cos}\;L\;\text{sin}\;\omega t\\ \omega_{ie}\text{cos}\;L\;\text{cos}\;\omega t\\ \omega_{ie}\text{sin}\;L+\omega \end{array}\right]$$ (7)

$$\delta \omega_{SF}^{s}=\left[\begin{array}{ccc} K_{gx} & 0 & 0\\ 0 & K_{gy} & 0\\ 0 & 0 & K_{gz} \end{array}\right]\omega_{is}^{s}\text{=}\left[\begin{array}{c} K_{gx}\omega_{ie}\text{cos}\;L\;\text{sin}\;\omega t\\ K_{gy}\omega_{ie}\text{cos}\;L\;\text{cos}\;\omega t\\ K_{gz}\left(\omega_{ie}\text{sin}\;L+\omega \right) \end{array}\right]$$ (8)

where *ω_ie_* is the rotational angular velocity generated by the earth’s rotation, $\omega_{is}^{s}$ is the theoretical rotational angular velocity provided by the gyroscope under the IMU frame, *L* is the latitude, $\delta \omega_{SF}^{s}$ is the sensor error triggered by the scale factor, *K*_*gx*_,*K*_*gy*_,*K*_*gz*_ are affected by the scale factor of the three-axis gyroscope.

The gyroscope error caused by scale factor in IMU coordinate system needs to be expressed in navigation coordinate system. Eq (9)) describes the transformation matrix of this process. Eq (10) gives the attitude error caused by full rotation.


$$\delta \omega_{SF+}^{n}=C_{b}^{n}C_{s}^{b}\delta \omega_{SF}^{s}=\left[\begin{array}{c} \left(K_{gx}-K_{gy}\right)\omega_{ie}\text{cos}\;L\;\text{sin}\;\omega t\;\text{cos}\;\omega t\\ \left(K_{gx}{\text{sin}}^{2}\omega t+K_{gy}{\text{cos}}^{2}\omega t\right)\omega_{ie}\text{cos}\;L\\ K_{gz}\left(\omega_{ie}\text{sin}\;L+\omega \right) \end{array}\right]$$ (9)

$$\int_{0}^{T}\delta \omega_{SF+}^{n}dt=\left[\begin{array}{c} 0\\ \frac{T\omega_{ie}\text{cos}\;L}{2}\left(K_{gx}+K_{gy}\right)\\ K_{gz}\left(\omega_{ie}\text{sin}\;L+\omega \right)T \end{array}\right]$$ (10)

The influence of the scale factor of the gyroscope will not produce azimuth error in a single rotation cycle. However, because the scale factors of the *x*_*s*_ axis and *y*_*s*_ axis are coupled with the rotation component in the north direction, the attitude error will increase with time in the north direction. At the same time, due to the close correlation between the influence of the scale factor of the *z*_*s*_ axis and the rotation frequency of the earth and the IMU, there will be an upward attitude error, namely heading error. This error is significant because the rotation rate of IMU is much higher than that of the earth. If the PPM is 10000 and the rotation speed is 6°/s, the IMU with unidirectional rotation can produce an attitude error of nearly 2 degrees in 30s. Therefore, a modulation scheme of reciprocating rotation is proposed in this paper. IMU will produce gyroscope error when it rotates in reverse direction due to the influence of scale factor, Eq (11) describes this process. The integral of the whole rotation error is shown in Eq (12).


$$\delta \omega_{SF-}^{n}=\left[\begin{array}{c} \left(-K_{gx}+K_{gy}\right)\omega_{ie}\text{cos}\;L\;\text{sin}\;\omega t\;\text{cos}\;\omega t\\ \left(K_{gx}{\text{sin}}^{2}\omega t+K_{gy}{\text{cos}}^{2}\omega t\right)\omega_{ie}\text{cos}\;L\\ K_{gz}\left(\omega_{ie}\text{sin}\;L-\omega \right) \end{array}\right]$$ (11)

$$\int_{0}^{T^{\prime }}\delta \omega_{SF}^{n}dt=\int_{0}^{T}\delta \omega_{SF+}^{n}dt+\int_{T}^{2T}\delta \omega_{SF-}^{n}dt=\left[\begin{array}{c} 0\\ T^{\prime }\omega_{ie}\text{cos}\;L\left(K_{gx}+K_{gy}\right)\\ T^{\prime }K_{gz}\omega_{ie}\text{sin}\;L \end{array}\right]$$ (12)

The scale factor error analysis of accelerometer is similar to that of gyroscope. When the IMU rotates around the *z*_*s*_ axis, the accelerometer cannot provide motion information, so only gravity information can be observed. Eq (13) gives the scale factor error of the accelerometer, while Eq (14) gives the integral of the entire rotation in the navigation frame.

$$\delta a_{SF}^{s}=\left[\begin{array}{ccc} K_{ax} & 0 & 0\\ 0 & K_{ay} & 0\\ 0 & 0 & K_{az} \end{array}\right]\left[\begin{array}{c} 0\\ 0\\ g \end{array}\right]\text{=}\left[\begin{array}{c} 0\\ 0\\ K_{az}g \end{array}\right]$$ (13)

$$\int_{0}^{T^{\prime }}\delta a_{SF}^{n}dt=\left[\begin{array}{c} 0\\ 0\\ T^{\prime }K_{az}g \end{array}\right]$$ (14)

where $\delta a_{SF}^{s}$ represents the scale factor error of the accelerometer under the IMU frame, $\delta a_{SF}^{n}$ represents the scale factor error of the accelerometer under the navigation frame, *K*_*ax*_,*K*_*ay*_,*K*_*az*_ represents the scale factor brought by the accelerometer on the three coordinate axes, and *g* represents gravity, but it should be noted that the above equation only needs to consider the local component of gravity in the vertical direction.

Similar to the situation of gyroscopes, the scale factor of the accelerometer on the horizontal plane will not produce any error after reciprocating rotation, but due to the correlation between the scale factor and gravity on the rotation axis, there is always a velocity error accumulated with time in the vertical direction.

#### Installation error

In theory, the three-axis accelerometer and gyroscope are installed on the IMU orthogonally. However, due to the installation error, the accelerometer and gyroscope are not orthogonal. This installation error will also cause attitude error. Eq (15) shows the gyroscope error caused by IMU installation error in the reciprocating rotation cycle.

$$\delta \omega_{N}^{s}=N\omega_{is}^{s}=\left[\begin{array}{ccc} 0 & K_{gxy} & K_{gxz}\\ K_{gyx} & 0 & K_{gyz}\\ K_{gzx} & K_{gzy} & 0 \end{array}\right]\left[\begin{array}{c} \omega_{ie}\text{cos}\;L\;\text{sin}\;\omega t\\ \omega_{ie}\text{cos}\;L\;\text{cos}\;\omega t\\ \omega_{ie}\text{sin}\;L+\omega \end{array}\right]$$ (15)

where *K*_*gij*_ represents the installation error parameters of the three coordinate axes, and $\delta \omega_{N}^{s}$ represents the influence amount of the gyroscope installation error. At the same time, due to the influence of three-axis installation error, *N* represents non-orthogonal matrix.

Eq (16) describes the gyroscope error caused by the installation error, and Eq (17) represents the whole circle rotation integration under the navigation frame [23].


$$\begin{array}{c} \delta \omega_{N}^{n}=C_{b}^{n}C_{s}^{b}\delta \omega_{N}^{s}\\ =\left[\begin{array}{c} \omega_{ie}\text{cos}\;L\left(K_{gxy}{\text{cos}}^{2}\omega t-K_{gyx}{\text{sin}}^{2}\omega t\right)+\left(\omega_{ie}\text{sin}\;L+\omega \right)\left(K_{gxz}\text{cos}\;\omega t-K_{gyz}\text{sin}\;\omega t\right)\\ \omega_{ie}\text{cos}\;L\;\text{sin}2\omega t\left(K_{gxy}+K_{gyx}\right)/2+\left(\omega_{ie}\text{sin}\;L+\omega \right)\left(K_{gxz}\text{sin}\;\omega t+K_{gyz}\text{cos}\;\omega t\right)\\ K_{gzx}\omega_{ie}\text{cos}\;L\;\text{sin}\;\omega t+K_{gzy}\omega_{ie}\text{cos}\;L\;\text{cos}\;\omega t \end{array}\right] \end{array}$$ (16)

$$\int_{0}^{T}\delta \omega_{N+}^{n}dt=\left[\begin{array}{c} \frac{T\omega_{ie}\text{cos}\;L}{2}\left(K_{gxy}-K_{gyx}\right)\\ 0\\ 0 \end{array}\right]$$ (17)

Eq (18) describes the influence of gyroscope installation error when IMU rotates in reverse direction under the navigation frame. The corresponding integral of the whole rotation error is shown in Eq (19).


$$\delta \omega_{N-}^{n}=\left[\begin{array}{c} \omega_{ie}\text{cos}\;L\left(K_{gxy}{\text{cos}}^{2}\omega t-K_{gyx}{\text{sin}}^{2}\omega t\right)+\left(\omega_{ie}\text{sin}\;L-\omega \right)\left(K_{gxz}\text{cos}\;\omega t+K_{gyz}\text{sin}\;\omega t\right)\\ -\omega_{ie}\text{cos}\;L\;\text{sin}2\omega t\left(K_{gxy}+K_{gyx}\right)/2+\left(\omega_{ie}\text{sin}\;L-\omega \right)\left(K_{gyz}\text{cos}\;\omega t-K_{gxz}\text{sin}\;\omega t\right)\\ -K_{gzx}\omega_{ie}\text{cos}\;L\;\text{sin}\;\omega t+K_{gzy}\omega_{ie}\text{cos}\;L\;\text{cos}\;\omega t \end{array}\right]$$ (18)

$$\int_{0}^{T^{\prime }}\delta \omega_{N}^{n}dt=\int_{0}^{T}\delta \omega_{N+}^{n}dt+\int_{T}^{2T}\delta \omega_{N-}^{n}dt=\left[\begin{array}{c} \omega_{ie}\text{cos}\;L(K_{gxy}-K_{gyx})T^{\prime }-2K_{gyz}\\ 2K_{gxz}\\ 0 \end{array}\right]$$ (19)

Similar to the gyroscope error analysis, Eq (20) describes the corresponding accelerometer error integration.

$$\int_{0}^{T^{\prime }}\delta a_{N}^{n}dt=\left[\begin{array}{c} T^{\prime }K_{axz}g\\ T^{\prime }K_{ayz}g\\ 0 \end{array}\right]$$ (20)

where $\delta a_{N}^{n}$ is the influence component of accelerometer installation error. At the same time, it can be seen that *K*_*axz*_ and *K*_*ayz*_ present gravity correlation in local areas, resulting in velocity errors in the east and north directions.

### 3.2. Simulation experiment and result analysis

In the previous section, the rotary modulation technology is introduced and three types of errors are analyzed. In order to study the effectiveness of the proposed scheme, a comparative experiment between the traditional strapdown system and the proposed rotary system is designed. Table 2 describes the environment and setting parameters of the simulation experiment. The following figures show the experimental results of attitude error caused by constant deviation, scale factor and axis misalignment.

**Table 2:** Simulation parameter configuration.

Simulation conditions	parameter
Navigation time	4 h
Latitude	42.4653° N
Sampling interval	10 ms
Rotation rate	30 deg/s
Gyroscope offset	10 deg/h random deviation+0.05 scale factor error
Accelerometer offset	10 mg deviation

**Fig 6:**
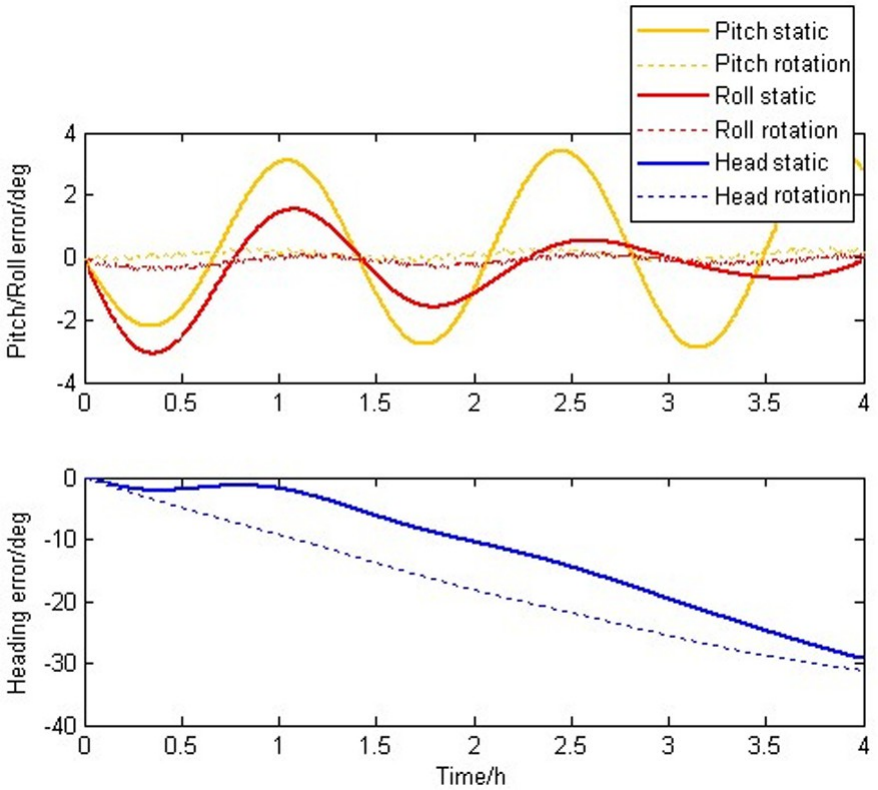
Attitude errors caused by constant bias of gyroscopes.

Fig 6 shows the attitude errors caused by constant bias of gyroscopes. During the four-hour experiment, we can be seen that the pitch and roll errors of the static IMU without rotation modulation oscillate in ±4 degrees and the pitch and roll errors of SIAMS modulated by rotation is reduced to ±0.1 degree. this result proves that the reciprocating rotation scheme proposed in this paper can automatically compensate the errors caused by constant bias of gyroscopes to some extent.

**Fig 7:**
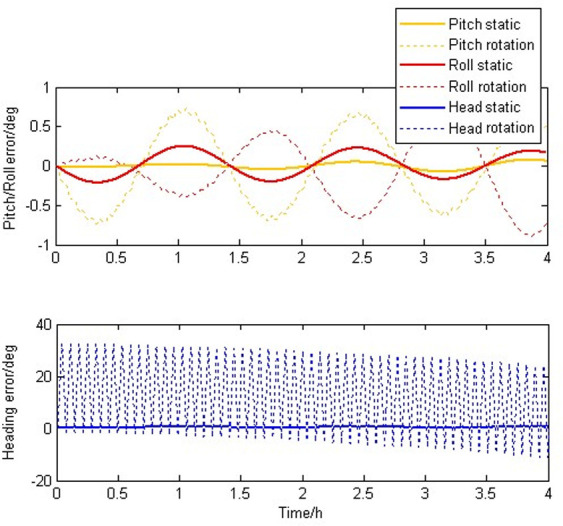
Attitude errors caused by scale factors of gyroscopes.

Fig 7 shows attitude errors due to scale factor asymmetry. during the four-hour experiment, it can be seen that the attitude errors of static IMU and SIAMS modulated by rotation oscillate greatly. For pitch and roll errors, the rotation-modulated SIAMS is more affected by scale factor asymmetry, while for heading errors, the static IMU exhibits divergent form and the rotation-modulated SIAMS exhibits oscillatory form.

**Fig 8:**
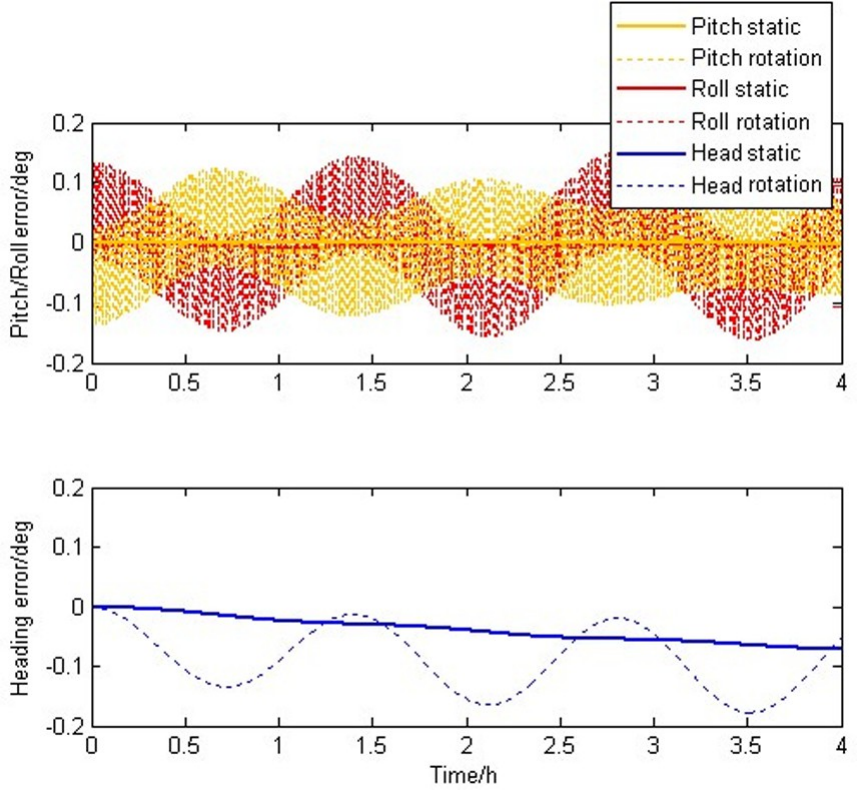
Attitude errors caused by the axis misalignment errors of the gyroscopes.

Fig 8 shows the attitude error caused by misalignment of the gyroscope axis. By observing the experimental results, it can be found that the static IMU generated a relatively small divergence error, while the SIAMS modulated by rotation generated a large oscillation error. This is because the SIAMS modulated by the reciprocating rotation method has a strong correlation between the misalignment of the gyroscope axis and the Earth’s rotation, resulting in a large divergence attitude error.

Through the above simulation experiments, we have come to a conclusion that SIAMS using a reciprocating rotation scheme can effectively suppress attitude errors caused by constant bias of gyroscopes. However, the scale factor and the axis misalignment of gyroscopes greatly affect the application of rotational modulation technology. Therefore, it is necessary to introduce an internal parameter calibration method based on SIAMS to eliminate the influence of gyroscope scale factor and axis misalignment, so that rotational modulation technology can provide better results.

## 4. Combine calibration method of multi position for MEMS-based rotary SIAMS

### 4.1. Modeling

The MEMS based rotational SIAMS can provide accurate attitude data. Through the modeling and analysis of noise, drift, scale factor and other errors, it can be known that the attitude accuracy is inversely proportional to time. In the previous section, through experimental analysis, it is clear that the rotary error modulation is effective to eliminate the errors of gyroscope and accelerometer, and it is also confirmed that the attitude error caused by the scale factor and installation error still affects the efficiency of the system. In order to solve this problem, we need to design a method to calibrate these errors. Common IMU calibration methods are mostly used for tactical-grade IMU and other high-quality sensors, and often need to introduce special references, such as preset frames or turntables [24–26]. Therefore, this paper presents a combine calibration method of multi position rotation without introducing special reference.

The IMU is rotated to eight different preset positions through the rotating device, and the output error of the Oz axis of SIAMS in the navigation frame of each position is calculated at the same time. Because the MEMS bias and scale factor error will cause different output errors in different positions, the MEMS drift and scale factor error can be calculated by combining the eight position SIAMS error output information based on the relationship between SIAMS error sources and output errors, and the error calibration task can be completed finally.

Eq (21) modeling the navigation error of SIAMS gyroscope,

$$\left[\begin{array}{c} N_{gx}\\ N_{gy}\\ N_{gz} \end{array}\right]=\left[\begin{array}{ccc} K_{gx} & K_{gxy} & K_{gxz}\\ K_{gyx} & K_{gy} & K_{gyz}\\ K_{gzx} & K_{gzy} & K_{gz} \end{array}\right]\left[\begin{array}{c} \omega_{x}\\ \omega_{y}\\ \omega_{z} \end{array}\right]+\left[\begin{array}{c} D_{x}\\ D_{y}\\ D_{z} \end{array}\right]$$ (21)

where *N*_*gx*_,*N*_*gy*_,*N*_*gz*_ represent the genuine output of the gyroscope in *x*, *y* and *z*, *D*_*x*_,*D*_*y*_,*D*_*z*_ represent the gyroscope bias in *x*, *y* and *z*.

**Fig 9:**
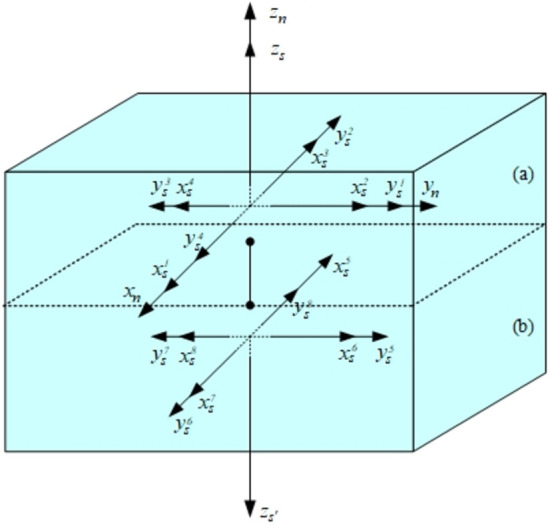
The position for MEMS calibration.

The original position of IMU is shown in Fig 9(A) (*x*_*n*_,*y*_*n*_,*z*_*n*_,), and the IMU frame coincides with the navigation frame in the original state, that is, $\left(x_{s}^{1},y_{s}^{1},z_{s}\right)$ coincides with (*x*_*n*_,*y*_*n*_,*z*_*n*_). Apply the transformation matrix from the *s*-frame to the *n-*frame for 8 different positions $\left(x_{s}^{i},y_{s}^{i},z_{s}{\text{/z}}_{s}^{'}\;i=1,2,\ldots \ldots ,8\right)$. Its corresponding gyroscope error is represented by formula (22–25).

$$\left\{\begin{array}{l} \delta N_{gx1\text{3}}=N_{gx1}+N_{gx\text{3}}=-2K_{gxy}(C_{32}\omega_{ie}\text{cos}\;L+C_{33}\omega_{ie}\text{sin}\;L)+2D_{x}\\ \delta N_{gy1\text{3}}=N_{gy1}+N_{gy\text{3}}=2K_{gyx}(C_{32}\omega_{ie}\text{cos}\;L+C_{33}\omega_{ie}\text{sin}\;L)+2D_{y}\\ \delta N_{gz1\text{3}}=N_{gz1}+N_{gz\text{3}}=2K_{gz}(C_{32}\omega_{ie}\text{cos}\;L+C_{33}\omega_{ie}\text{sin}\;L)+2D_{z} \end{array}\right.$$ (22)

$$\left\{\begin{array}{l} \delta N_{gx\text{57}}=N_{gx\text{5}}+N_{gx\text{7}}=2K_{gxy}(C_{32}\omega_{ie}\text{cos}\;L+C_{33}\omega_{ie}\text{sin}\;L)+2D_{x}\\ \delta N_{gy\text{57}}=N_{gy\text{5}}+N_{gy\text{7}}=-2K_{gyx}(C_{32}\omega_{ie}\text{cos}\;L+C_{33}\omega_{ie}\text{sin}\;L)+2D_{y}\\ \delta N_{gz\text{57}}=N_{gz\text{5}}+N_{gz\text{7}}=-2K_{gz}(C_{32}\omega_{ie}\text{cos}\;L+C_{33}\omega_{ie}\text{sin}\;L)+2D_{z} \end{array}\right.$$ (23)

$$\left\{\begin{array}{l} \delta N_{gx\text{26}}=N_{gx\text{2}}+N_{gx\text{6}}=-2K_{gxz}(C_{12}\omega_{ie}\text{cos}\;L+C_{13}\omega_{ie}\text{sin}\;L)+2D_{x}\\ \delta N_{gy\text{26}}=N_{gy\text{2}}+N_{gy\text{6}}=-2K_{gy}(C_{12}\omega_{ie}\text{cos}\;L+C_{13}\omega_{ie}\text{sin}\;L)+2D_{y}\\ \delta N_{gz\text{26}}=N_{gz\text{2}}+N_{gz\text{6}}=2K_{gzx}(C_{12}\omega_{ie}\text{cos}\;L+C_{13}\omega_{ie}\text{sin}\;L)+2D_{z} \end{array}\right.$$ (24)

$$\left\{\begin{array}{l} \delta N_{gx\text{48}}=N_{gx\text{4}}+N_{gx\text{8}}=2K_{gxz}(C_{12}\omega_{ie}\text{cos}\;L+C_{13}\omega_{ie}\text{sin}\;L)+2D_{x}\\ \delta N_{gy\text{48}}=N_{gy\text{4}}+N_{gy\text{8}}=2K_{gy}(C_{12}\omega_{ie}\text{cos}\;L+C_{13}\omega_{ie}\text{sin}\;L)+2D_{y}\\ \delta N_{gz\text{48}}=N_{gz\text{4}}+N_{gz\text{8}}=-2K_{gzx}(C_{12}\omega_{ie}\text{cos}\;L+C_{13}\omega_{ie}\text{sin}\;L)+2D_{z} \end{array}\right.$$ (25)

where *C*_*ij*_ is the element of transformation matrix $C_{s}^{n}$. The error parameters of SIAMS gyroscope can be calculated by combining the relationship of Eqs (22–25).


$$\left\{\begin{array}{l} D_{x}=\frac{\delta N_{gx1\text{3}}+\delta N_{gx\text{57}}}{4}\\ D_{y}=\frac{\delta N_{gy1\text{3}}+\delta N_{gy\text{57}}}{4}\\ D_{z}=\frac{\delta N_{gz1\text{3}}+\delta N_{gz\text{57}}}{4} \end{array}\right.$$ (26)

$$\left\{\begin{array}{l} K_{gxy}=-\frac{\delta N_{gx1\text{3}}-\delta N_{gx\text{57}}}{4(C_{32}\omega_{ie}\text{cos}\;L+C_{33}\omega_{ie}\text{sin}\;L)}\\ K_{gyx}=\frac{\delta N_{gy1\text{3}}-\delta N_{gy\text{57}}}{4(C_{32}\omega_{ie}\text{cos}\;L+C_{33}\omega_{ie}\text{sin}\;L)}\\ K_{gz}=\frac{\delta N_{gz1\text{3}}-\delta N_{gz\text{57}}}{4(C_{32}\omega_{ie}\text{cos}\;L+C_{33}\omega_{ie}\text{sin}\;L)} \end{array}\right.$$ (27)

$$\left\{\begin{array}{l} K_{gxz}=-\frac{\delta N_{gx\text{26}}-\delta N_{gx\text{48}}}{4(C_{12}\omega_{ie}\text{cos}\;L+C_{13}\omega_{ie}\text{sin}\;L)}\\ K_{gy}=-\frac{\delta N_{gy\text{26}}-\delta N_{gy\text{48}}}{4(C_{12}\omega_{ie}\text{cos}\;L+C_{13}\omega_{ie}\text{sin}\;L)}\\ K_{gzx}=\frac{\delta N_{gz\text{26}}-\delta N_{gz\text{48}}}{4(C_{12}\omega_{ie}\text{cos}\;L+C_{13}\omega_{ie}\text{sin}\;L)} \end{array}\right.$$ (28)

Combining Eq (28) with the output equations of level gyroscopes:

$$\left\{\begin{array}{l} \delta N_{gx\text{26}}-\delta N_{gx\text{48}}=-4K_{gxz}(C_{12}\omega_{ie}\text{cos}\;L+C_{13}\omega_{ie}\text{sin}\;L)\\ \delta N_{gy\text{26}}-\delta N_{gy\text{48}}=-4K_{gy}(C_{12}\omega_{ie}\text{cos}\;L+C_{13}\omega_{ie}\text{sin}\;L)\\ \delta N_{gz\text{26}}-\delta N_{gz\text{48}}=4K_{gzx}(C_{12}\omega_{ie}\text{cos}\;L+C_{13}\omega_{ie}\text{sin}\;L)\\ N_{gx1}=K_{gx}(C_{12}\omega_{ie}\text{cos}\;L+C_{13}\omega_{ie}\text{sin}\;L)+K_{gxz}(C_{22}\omega_{ie}\text{cos}\;L+C_{23}\omega_{ie}\text{sin}\;L)-K_{gxy}(C_{32}\omega_{ie}\text{cos}\;L+C_{33}\omega_{ie}\text{sin}\;L)+D_{x}\\ N_{gy1}=-K_{gyz}(C_{12}\omega_{ie}\text{cos}\;L+C_{13}\omega_{ie}\text{sin}\;L)+K_{gy}(C_{22}\omega_{ie}\text{cos}\;L+C_{23}\omega_{ie}\text{sin}\;L)+K_{gyx}(C_{32}\omega_{ie}\text{cos}\;L+C_{33}\omega_{ie}\text{sin}\;L)+D_{x}\\ N_{gx2}=-K_{gxz}(C_{12}\omega_{ie}\text{cos}\;L+C_{13}\omega_{ie}\text{sin}\;L)+K_{gx}(C_{22}\omega_{ie}\text{cos}\;L+C_{23}\omega_{ie}\text{sin}\;L)-K_{gxy}(C_{32}\omega_{ie}\text{cos}\;L+C_{33}\omega_{ie}\text{sin}\;L)+D_{x}\\ N_{gy2}=-K_{gy}(C_{12}\omega_{ie}\text{cos}\;L+C_{13}\omega_{ie}\text{sin}\;L)-K_{gyz}(C_{22}\omega_{ie}\text{cos}\;L+C_{23}\omega_{ie}\text{sin}\;L)+K_{gyx}(C_{32}\omega_{ie}\text{cos}\;L+C_{33}\omega_{ie}\text{sin}\;L)+D_{x} \end{array}\right.$$ (29)

where *C*_32_*ω*_*N*_+*C*_33_*ω*_*U*_ is a constant when the vehicle is in static. For this reason, knowing from Eqs (26)–(29), we can get the eleven error parameters for SIAMS. Substituting these known error parameters into Eq (21), the last error parameter *K*_*gzy*_ can be calculated, as shown in Eq (30):

$$K_{gzy}=\frac{N_{gz1}+K_{gzx}(C_{22}\omega_{ie}\text{cos}\;L+C_{23}\omega_{ie}\text{sin}\;L)-K_{gz}(C_{32}\omega_{ie}\text{cos}\;L+C_{33}\omega_{ie}\text{sin}\;L)-D_{z}}{C_{12}\omega_{ie}\text{cos}\;L+C_{13}\omega_{ie}\text{sin}\;L}$$ (30)

### 4.2. Experiments and analyses

A combined calibration method of multi position rotation is verified by rotating the SIAMS fixed on the rotating device to different positions. The performance indexes of the rotating device are shown in Table 3.

**Table 3:** The main parameters of rotating device.

*Load requirements*	40kg	*Position accuracy*	±3″
*Rotary precision*	Inner Middle Outer Gimbal (±4″)	*Operating mode*	Idle Location Rate Servo rocking
*Angle measurement accuracy*	±3″	*Angle measurement repetition*	±3″
*Rate requirements*	0.0001°-100°/s	*Rate accuracy*	1×10^−5^ (mean of 360°) 1×10^−3^ (mean of 1°)

Fix SIAMS on the rotating device and rotate according to the track described in Fig 9. The rest time of eight positions is 20 minutes. Attitude reference is provided by SPAN-LCI produced by NovAtel Company in Canada. The attitude error based on GPS assistance is less than 0.025 degrees.

In order to comprehensively analyze the effectiveness of the proposed scheme, the experiment sets up two situations, one is conducted under the condition of slow change of heading, and the other is conducted under the condition of sudden change of heading.

**Fig 10:**
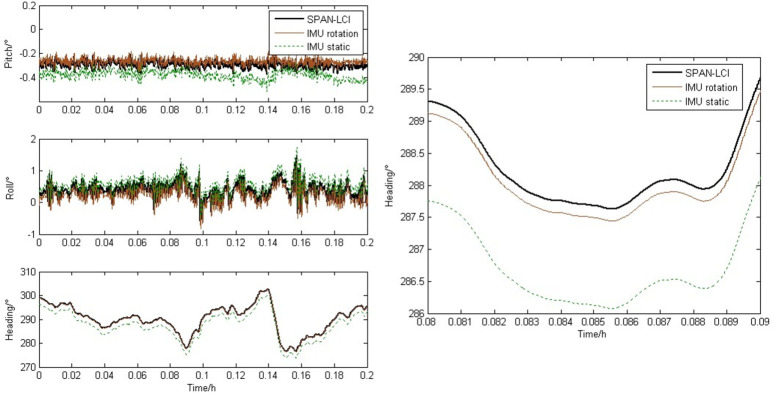
Curves of attitude error with heading changed slowly. (a) Attitude overall view. (b) Heading partial enlarged view.

Fig 10 shows the comparison curve of attitude information under slowly changing heading conditions. A comprehensive analysis of Fig 10(A) and 10(B) shows that the attitude curve of the rotating strapdown inertial navigation system is more accurate than that of the static inertial navigation system. Taking the heading angle as an example, it can be seen from Fig 10(B) that the maximum amplitude error has decreased from 1.5 degrees to 0.2 degrees.

**Fig 11:**
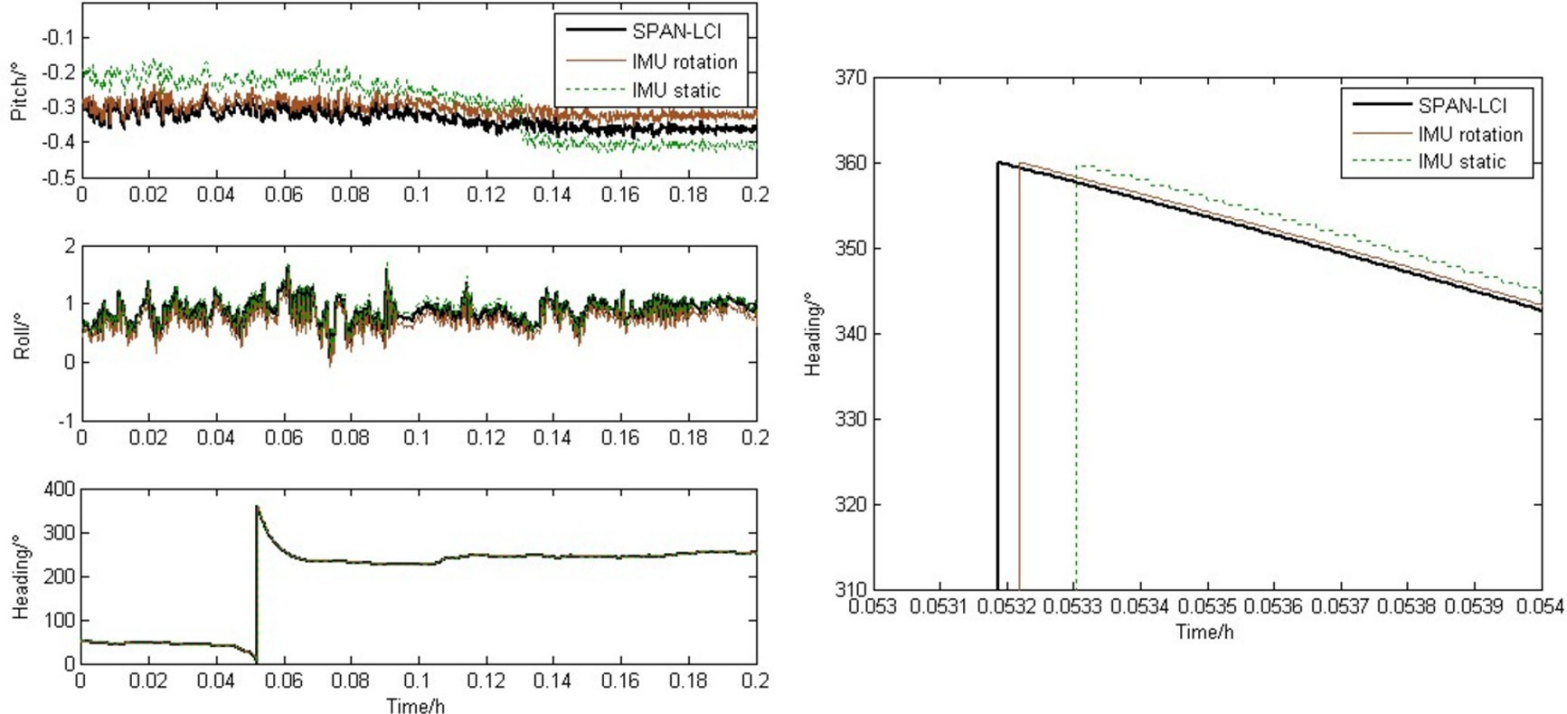
Curves of attitude error with heading changed abruptly. (a) Attitude overall view. (b) Heading partial enlarged view.

Fig 11 shows the comparison curve of attitude information under sudden changes in heading conditions. By comprehensively analyzing Fig 11(A) and 11(B), it can be found that sudden applied heading changes do not affect the accuracy of the attitude curve provided by the rotating strapdown inertial navigation system. Taking the heading angle as an example, it can be seen from Fig 11(B) that the maximum amplitude error has decreased from 2.5 degrees to 0.35 degrees. Therefore, it can be considered that after using the multi position rotation joint calibration method for error calibration, the attitude error (especially the heading error) has been greatly weakened. This improves the IMU attitude measurement accuracy of the reciprocating rotation scheme once further.

## 5. Conclusions

MEMS inertial sensors have been widely used because of their light weight, low cost and other advantages, but the accuracy level is far lower than that of high grade inertial sensors. This makes the research on modulation and calibration of MEMS errors very important. In this paper, a MEMS strapdown inertial attitude measurement system using rotating modulation technology is proposed. It realizes the automatic compensation of sensor error through rotating inertial measurement unit. It provides a new idea for MEMS to achieve the accuracy of high grade inertial sensors. The validity of the proposed method is proved by the analysis and experiment of three kinds of sensor deterministic errors: constant bias, scale factor and installation error. Analysis and simulation results can be drawn as follows.

1. The rotary modulation technology can further compensate the significant error when the IMU rotates relative to the navigation coordinate system, especially the error caused by constant bias. However, the scale factor error and axis misalignment error of MEMS may be coupled with the Earth’s rotation, resulting in certain long-term navigation errors. At the same time, it was found that for SIAMS with one rotating axis, the misalignment error of the axis has the greatest impact on the navigation position.
2. The proposed multi position rotation combined calibration method can significantly reduce attitude errors, especially heading errors. The combination of rotational modulation technology and multi position calibration method can greatly improve the accuracy of IMU attitude measurement.

This article discusses the rotation scheme of SIAMS, providing a new approach for using low-cost equipment to provide the attitude and position accuracy of tactical-grade IMU.

## References

[1] Fu H P, Cheng Y M. Switching Gaussian-heavy-tailed distribution based robust Gaussian approximate filter for INS/GNSS integration[J]. Journal of the Franklin Institute, 2022, 359(16):9271–9295.

[2] Sun W, Liu J Z. Design of robust cubature fission particle filter algorithm in multi-source cooperative navigation[J]. Scientific Reports, 2022, 12(1): 4210–4210. 35273318 10.1038/s41598-022-08189-x PMC8913726

[3] Wu G, Fang X Q, Song Y, et al. Study on the Shearer Attitude Sensing Error Compensation Method Based on Strapdown Inertial Navigation System[J]. Applied Sciences, 2022, 12(21): 10848–10848.

[4] Ding P, Cheng X H. A new Contour-Based combined matching algorithm for underwater Terrain-Aided strapdown inertial navigation system[J]. Measurement, 2022, 202.

[5] Zhang F B, Gao X H, Song W B, et al. A Vision Aided Initial Alignment Method of Strapdown Inertial Navigation Systems in Polar Regions[J]. Sensors, 2022, 22(13): 4691–4691. 35808188 10.3390/s22134691 PMC9268847

[6] Wei S, Yang G. Fiber-based rotary strapdown inertial navigation system[J]. Optical Engineering, 2013, 52(7): 076106–076106.

[7] Wang M, Wang L. Influence of gyro axis sequence configuration on the accuracy of alignment and navigation in a nested rotation inertial navigation system[J]. Measurement Science and Technology, 2022, 33(11).

[8] Yuriy P, Dmitriy B, Irina H, et al. Closed-form quaternion representations for rigid body rotation: application to error assessment in orientation algorithms of strapdown inertial navigation systems[J]. Continuum Mechanics and Thermodynamics, 2020: 1–20.

[9] Xueyun W, Jie W, Tao X, et al. Analysis and Verification of Rotation Modulation Effects on Inertial Navigation System based on MEMS Sensors[J]. Journal of Navigation, 2013, 66(5): 751–772.

[10] Du S. A micro-electro-mechanical-system-based inertial system with rotating accelerometers and gyroscopes for land vehicle navigation[J]. Int. J. Distrib. Sens. Netw. 2017, 13, 1550147717746351.

[11] Jing Z, Li J, Zhang X, et al. A Novel Rotation Scheme for MEMS IMU Error Mitigation Based on a Missile-Borne Rotation Semi-Strapdown Inertial Navigation System[J]. Sensors 2019, 19, 1683. 30970555 10.3390/s19071683 PMC6479492

[12] Wang Z S, Wang X S, Zhao Y W, et al. Research on Integrated Navigation and Positioning Technology of Inertial Navigation System and Odometer Based on Factor Graph[C]. Advances in Precision Instruments and Optical Engineering, 2022,:467–475.

[13] Soni R, Trapasiya S. A survey of step length estimation models based on inertial sensors for indoor navigation systems[J]. International Journal of Communication Systems, 2021, 35(4).

[14] Chen W N, Yang Z, Gu S S, et al. Adaptive transfer alignment method based on the observability analysis for airborne pod strapdown inertial navigation system[J]. Scientific Reports, 2022, 12(1): 946–946. 35042924 10.1038/s41598-021-04732-4 PMC8766501

[15] Seo Y B, Yu H, Ryu K, et al. Analysis of Gyro Bias Depending on the Position of Inertial Measurement Unit in Rotational Inertial Navigation Systems[J]. Sensors, 2022, 22(21): 8355–8355. 36366052 10.3390/s22218355 PMC9653864

[16] Wei Q S, Zha F, He H Y, et al. An Improved System-Level Calibration Scheme for Rotational Inertial Navigation Systems[J]. Sensors, 2022, 22(19): 7610–7610. 36236706 10.3390/s22197610 PMC9571831

[17] Zha F, Chang L B, He H Y, et al. Comprehensive Error Compensation for Dual-Axis Rotational Inertial Navigation System[J]. IEEE Sensors Journal, 2020, 20(7): 3788–3802.

[18] Hao H, Lei W, Wang M. An Online Gyro Scale Factor Error Calibration Method for Laser RINS[J]. IEEE Sensors Journal, 2021, 21(13): 15291–15298.

[19] Fei Y, Qian S. Angular Rate Optimal Design for the Rotary Strapdown Inertial Navigation System[J]. Sensors, 2014, 14(4): 7156–7180. 24759115 10.3390/s140407156 PMC4029711

[20] Li Q H, Li K, Liang W W. A Dual-Axis Rotation Scheme for Long-Endurance Inertial Navigation Systems[J]. IEEE Transactions on Instrumentation and Measurement, 2022, 71(8503510): 1–10, 2022.

[21] Di W, Bing W, Huang H Q. Online Calibration Method of DVL Error, Based on Improved Integrated Navigation Model[J]. IEEE Sensors Journal, 2022, 22(21): 21082–21092.

[22] Li Q, Liu L, Ma X F, et al. Development of Multitarget Acquisition, Pointing, and Tracking System for Airborne Laser Communication[J]. IEEE Transactions on Industrial Informatics, 2019, 15(3):1720–1729.

[23] Wei Q S, Zha F, Chang L B, et al. Novel rotation scheme for dual-axis rotational inertial navigation system based on body diagonal rotation of inertial measurement unit[J]. Measurement Science and Technology, 2022, 33(9).

[24] Afonin A A, Sulakov A S, Maamo M S, et al. The development and evaluation of a combined initial alignment algorithm for strapdown inertial navigation system[J]. Journal of Physics: Conference Series, 2022, 2373(7).

[25] Wang Z H, Cheng X H. Adaptive optimization online IMU self-calibration method for visual-inertial navigation systems[J]. Measurement, 2021, 180.

[26] Li J, Su L C, Wang F, et al. An Improved Online Fast Self-Calibration Method for Dual-Axis RINS Based on Backtracking Scheme[J]. Sensors, 2022, 22(13): 5036–5036. 35808540 10.3390/s22135036 PMC9269788

